# Cucurbitacin B suppresses GBM progression by inducing GADD45α-Dependent PCNA downregulation and DNA replication arrest

**DOI:** 10.3389/fphar.2026.1804502

**Published:** 2026-10-14

**Authors:** Yonghua Xue, Bin Wang

**Affiliations:** Department of Neurosurgery, Putuo Hospital, Shanghai University of Traditional Chinese Medicine, Shanghai, China

**Keywords:** cell cycle arrest, Cucurbitacin B, GADD45α, glioblastoma, PCNA

## Abstract

**Background:**

Glioblastoma (GBM) is the most aggressive primary brain tumor, characterized by uncontrolled proliferation and resistance to current standard chemoradiotherapy. Natural products have emerged as promising anticancer agents, yet the therapeutic mechanisms of Cucurbitacin B (CuB), a highly bioactive triterpenoid, in GBM remain insufficiently understood. Our preliminary transcriptomic analysis revealed that CuB markedly upregulates GADD45α while suppressing PCNA, suggesting a previously unrecognized replication-stress regulatory axis.

**Purpose:**

This study aimed to determine the anti-GBM efficacy of CuB across bulk and stem-like tumor models and to elucidate the molecular cascade connecting GADD45α induction to PCNA-mediated DNA replication arrest.

**Methods:**

The anti-tumor effects of CuB were evaluated in GBM cell lines (LN229, U87) and patient-derived glioma stem cells (GSCs) via functional assays, flow cytometry, and extreme limiting dilution assays. Transcriptome sequencing guided mechanistic exploration. Gain- and loss-of-function assays (GADD45α knockdown/overexpression, PCNA overexpression) elucidated the signaling cascade. Therapeutic efficacy was assessed in an orthotopic GBM xenograft mouse model.

**Results:**

CuB significantly suppressed the malignant phenotypes of GBM cells and impaired the self-renewal capacity of GSCs, concomitantly inducing apoptosis and G2/M-phase arrest, as evidenced by Cyclin B1 suppression and CDK1 inhibitory phosphorylation. Mechanistically, CuB strongly upregulated GADD45α, which maintained the Retinoblastoma (Rb) protein in a hypophosphorylated, active state. Active Rb subsequently sequestered the E2F1 transcription factor, leading to the profound transcriptional downregulation of PCNA. Knockdown of GADD45α restored Rb/E2F1-driven PCNA expression and reversed CuB-mediated replication arrest, whereas GADD45α overexpression phenocopied CuB. Ectopic PCNA expression counteracted these suppressive effects. *In vivo*, CuB effectively reduced intracranial tumor burden and prolonged survival in a GADD45α-dependent manner, while exhibiting a highly favorable systemic safety profile in healthy mice.

**Conclusion:**

CuB suppresses GBM progression by activating GADD45α, which targets the Rb/E2F1 signaling axis to transcriptionally repress PCNA-mediated DNA replication. These findings highlight the GADD45α-Rb/E2F1-PCNA cascade as a critical mechanistic vulnerability and position CuB as a safe, promising replication-modulating agent for highly heterogeneous GBM.

## Introduction

Glioblastoma (GBM) is the most prevalent and aggressive primary malignant brain tumor in adults. Despite multimodal treatment strategies including maximal surgical resection, radiotherapy, and temozolomide-based chemotherapy (Bikfalvi et al., 2023; Jackson et al., 2019; Liu et al., 2024). The prognosis of patients with GBM remains poor, with a median survival of approximately 15 months. The highly infiltrative growth pattern, remarkable cellular heterogeneity, and resistance to conventional therapies contribute to the limited efficacy of current treatments (Bagley et al., 2025; Qin et al., 2018; Stupp et al., 2005). Therefore, identifying novel therapeutic targets and developing effective treatment strategies for GBM remain urgent clinical challenges.

Natural products represent an important source of anticancer drug discovery due to their diverse chemical structures and multi-target pharmacological activities (Ismail et al., 2023; Melfi et al., 2023; Naeem et al., 2022; Jiang et al., 2010; Song et al., 2024; Zheng et al., 2024). Among these compounds, Cucurbitacin B (CuB), a highly oxygenated tetracyclic triterpenoid isolated from plants of the Cucurbitaceae family, has demonstrated potent anticancer activities in multiple tumor types (Wang Z. et al., 2017; Yin et al., 2025; Zhang et al., 2025). Previous studies have shown that CuB suppresses tumor progression through several molecular mechanisms, including disruption of the actin cytoskeleton (Zhang et al., 2011; Yin et al., 2008; Li et al., 2024), inhibition of integrin-mediated cell adhesion (Touihri-Barakati et al., 2017), and modulation of signaling pathways such as JAK/STAT3 (Lv et al., 2025), GRP78-FOXM1-KIF20A pathway (Wei et al., 2022), and CIP2A-PP2A-Akt signaling (Qin et al., 2018). These mechanisms collectively contribute to the inhibition of tumor cell proliferation, migration, and survival. However, the molecular mechanisms underlying the anti-GBM activity of CuB remain incompletely understood.

Dysregulation of DNA replication and replication stress represents a central hallmark of cancer and plays a central role in uncontrolled tumor cell proliferation (Naim, 2024). Growth Arrest and DNA Damage-Inducible α (GADD45α) is a stress-responsive protein involved in DNA repair, cell-cycle checkpoint control, and replication stress signaling (Palomer et al., 2024). Increasing evidence suggests that GADD45α family proteins function as tumor suppressors in glioma (Hu et al., 2024; Shen et al., 2025). Conversely, proliferating cell nuclear antigen (PCNA) is a key sliding clamp protein that coordinates DNA replication and repair processes (Strzalka and Ziemienowicz, 2011), and its overexpression is closely associated with high-grade glioma and poor prognosis (Cai et al., 2021).

Based on transcriptomic profiling of CuB-treated GBM cells, we observed a marked upregulation of GADD45α accompanied by downregulation of PCNA, suggesting that CuB may interfere with replication-associated processes. We therefore hypothesized that CuB suppresses GBM progression by activating GADD45α and subsequently inhibiting PCNA-mediated DNA replication. In this study, we systematically investigated the anti-tumor effects of CuB in GBM cells and orthotopic mouse models and further explored the role of the GADD45α-PCNA axis in mediating its therapeutic activity.

## Materials and methods

### Chemicals and reagents

Cucurbitacin B (HY-N0416, purity >99.8%) was purchased from MedChemExpress (Shanghai, China). Primary antibodies against GADD45α (13747-1-AP), PCNA (10205-2-AP), CDK1 (19532-1-AP), p-CDK1 (82751-5-RR), E2F1 (12171-1-AP) and GAPDH was obtained from Proteintech (Wuhan, China). The antibody for Cleaved Caspase-3 (#9661), Cyclin B1(#4138), and p-Rb (#8516) was obtained from Cell Signaling Technology (Danvers, MA, USA). Fetal bovine serum (FBS), B27 supplement, epidermal growth factor (EGF), and basic fibroblast growth factor (bFGF) was purchased from Gibco (Thermo Fisher Scientific), while DMEM and other cell culture reagents were acquired from KenGEN Biotech (China). Furthermore, commercial biochemical assay kits used for the *in vivo* toxicity assessment, including alanine aminotransferase (ALT), aspartate aminotransferase (AST), blood urea nitrogen (BUN), and creatinine, were purchased from Nanjing Jiancheng Bioengineering Institute (Nanjing, China). Unless otherwise indicated, all additional chemicals and reagents were obtained from Sigma-Aldrich (St. Louis, MO, USA) and were of analytical grade.

### Mice

BALB/c-Nude mice (male, 5–6°weeks old) were obtained from the Model Animal Research Center of Shanghai University of Traditional Chinese Medicine. Animals were maintained under specific pathogen-free (SPF) conditions (temperature 21 °C ± 1 °C, relative humidity 50% ± 10%, 12 h light-dark cycle) with free access to food and water. For intracranial glioma xenograft experiments, all procedures were performed in accordance with the National Institutes of Health (NIH) Guide for the Care and Use of Laboratory Animals and were approved by the Institutional Animal Care and Use Committee (IACUC) of Shanghai University of Traditional Chinese Medicine (Approval No. IACUC-2406028). Humane endpoints, including the emergence of neurological deficits or >20% body weight loss, were strictly followed rather than allowing tumors to reach maximal burden. All surgical and experimental procedures were conducted under aseptic conditions to minimize animal suffering.

### Cell culture

Human GBM cell line LN229 and U87 were obtained from Shanghai Zhong Qiao Xin Zhou Biotechnology Co., Ltd (Shanghai, China). Cells were maintained in DMEM supplemented with 10% fetal bovine serum (FBS) and cultured at 37 °C in a humidified atmosphere containing 5% CO_2_.

Patient-derived glioma stem cells (GSCs) were obtained from Beijing IDMO Co. Ltd. (Beijing, China). GSCs were maintained as free-floating neurospheres in serum-free DMEM/F12 medium (Gibco) supplemented with 2% B27 (Gibco), 20 ng/mL EGF and 20 ng/mL bFGF. GSCs were cultured at 37 °C with 5% CO_2_, and half of the culture medium was replaced every 2–3 days. For passaging and subsequent functional assays, the neurospheres were enzymatically dissociated into single-cell suspensions using Accutase.

To generation stable luciferase-expressing GBM cells, LN229 and U87 were seeded in 6-well plates at a density of 1 × 10^6^ cells/well and transduced with LV-Luciferase virus (2 μL/well, 1 × 10^8^ TU/mL; GeneChem, Shanghai, China) in serum-free medium for 72 h. Following transduction, cells were transferred to complete medium and subjected to puromycin selection (2 μg/mL for 48 h) to eliminate non-transduced cells. Luciferase activity was quantified using the Bright-Glo Luciferase Assay System (Promega, E2620) and measured on a Varioskan LUX multimode microplate reader (Thermo Fisher Scientific). Cell populations demonstrating >100-fold luminescence above background were considered stably transduced and were expanded for subsequent experiments. All lentiviral procedures were conducted in a certified BSL-2 facility following institutional biosafety guidelines. Authentication of transduced cell lines was performed using short tandem repeat (STR) profiling, and cells were subjected to monthly *mycoplasma* contamination testing throughout the study.

Stable knockdown of GADD45α in LN229 and U87 cells was established using lentiviral shRNA. Lentiviral particles encoding GADD45α-targeting shRNA (LV-GADD45α shRNA, L26921) or a corresponding non-targeting control (LV-Control shRNA, L00015) were obtained from Beyotime Biotechnology. GBM cells (5 × 10^5^ per well) were plated in 6-well plates and allowed to reach 40%–50% confluence prior to infection. Cells were then exposed to LV-GADD45α shRNA or LV-control shRNA virus at a Multiplicity of Infection (MOI) of 20 for 24 h. After viral incubation, the medium was replaced with fresh complete culture medium and cultures were maintained for an additional 48 h. To establish stable cell populations, transduced LN229 and U87 cells were treated with puromycin (2 μg/mL) for 7–10 days, with medium replenished every 48 h until all non-infected control cells were eliminated. Surviving populations were expanded to generate polyclonal GADD45α-knockdown stable cell lines.

For overexpression studies, recombinant AAV vectors encoding GADD45α (AAV-GADD45α), PCNA (AAV-PCNA), EGFP control (AAV-EGFP) or mCherry control (AAV-mCherry) were purchased from Obio Technology (Shanghai, China). LN229 and U87 cells were seeded in 6-well plates at 5 × 10^5^ cells per well and transduction was initiated when cultures reached approximately 50% confluence. Cells were incubated with the respective AAV preparations for 24 h, after which the viral supernatant was replaced with fresh complete medium and maintained for 72 h. Stable overexpressing cell populations were subsequently selected using puromycin, and surviving cells were expanded for following assays.

### Extreme limiting dilution assays (ELDA)

Single-cell suspensions of GSCs were seeded into ultra-low attachment 96-well plates at decreasing densities (e.g., 100, 50, 20, 10, and 1 cell per well), with 10 to 12 replicate wells for each cell density. The cells were then exposed to CuB or vehicle. After 10 to 14 days of continuous incubation, the number of wells containing at least one neurosphere (diameter >50 μm) was recorded. The stem cell frequency and statistical significance between groups were calculated using the ELDA software platform provided by the Walter and Eliza Hall Institute of Medical Research (http://bioinf.wehi.edu.au/software/elda/).

### CCK-8 assay

Cell viability was evaluated using the Cell Counting Kit-8 (CCK-8) following the manufacturer’s protocol. GBM cells were seeded in 96-well plates at a density of 2000 cells per well for 24 h. Cells were subsequently exposed to a gradient of CuB concentrations (0, 0.01, 0.1, 1, 10, and 100 μM) for 48 h. After drug treatment, 10 μL of CCK-8 reagent was added to each well and incubated for 2 h at room temperature. Absorbance at 450 nm was recorded using a microplate reader (TECAN). Cell viability was calculated using the following formula: Cell viability = [(OD_Experiment group_ − OD_Blank_)/(OD_Control group_ − OD_Blank_)] × 100%).

### Colony formation assay

For clonogenic survival analysis, GBM cells were seeded into 6-well plates at a density of 500 cells per well and maintained at 37 °C with 5% CO_2_. After 24 h, GBM cells were exposed to 100 nM CuB and allowed to grow for 14 days. Culture medium was refreshed every 3 days. At the end of the incubation period, cells were gently washed with phosphate-buffered saline (PBS), fixed with 4% paraformaldehyde (PFA) for 20 min, and stained with 0.1% crystal violet for 10 min. Excess dye was removed with distilled water, and the plates were air-dried. Colonies were photographed using a multifunctional microplate reader Cytation 5.

### Wound healing assay

GBM cells were seeded into 6-well plates at a density of 1 × 10^6^ cells per well and cultured at 37 °C and 5% CO_2_ until reaching 90%–100% confluence. A linear wound was created across the cell monolayer using a 200-μL pipette tip, and detached cells were removed by gently washing the wells with PBS. Cells were then incubated with medium containing 100 nM CuB for 48 h. Images of the wound area were captured immediately after scratching (0 h) and again at 48 h. The extent of cell migration was quantified using ImageJ software. The migration area (%) was calculated as: Migration area (%) = [Open area_48 h_]/[Initial open area_0 h_] × 100%.

### Transwell invasion assay

The invasive capacity of GBM cells was assessed using 24-well Transwell chambers precoated with a diluted Matrigel mixture (DMEM: Matrigel = 8:1). A total of 1 × 10^4^ cells suspended in 100 μL serum-free DMEM were seeded into the upper chamber, while 800 μL of medium containing 20% FBS was added to the lower chamber as a chemoattractant. 10 min later, 100 nM CuB was added to the upper chamber, and the plates were incubated for 48 h at 37 °C. Subsequently, cells were fixed with 4% PFA for 20 min. The remaining Matrigel and non-invading cells on the upper surface of the membrane were gently removed with a cotton swab. Invaded cells on the lower surface were stained with 0.1% crystal violet and air-dried. Cell invasion was quantified by counting stained cells in five randomly selected microscopic fields under a light microscope.

### EdU staining

GBM cells were seeded on sterile glass coverslips in 12-well plates at a density of 1 × 10^5^ cells per well and maintained in DMEM supplemented with 10% FBS and 1% penicillin-streptomycin. After 24 h of incubation, GBM cells were treated with or without 100 nM CuB for 48 h. For the final 2 h, 10 μM EdU was added to the culture medium to label actively proliferating cells. Following EdU incorporation, cells were fixed, permeabilized and stained using the Click-iT^TM^ EdU Alexa Fluor 488 kit according to the manufacturer’s protocol. Nuclei were counterstained with Hoechst. Fluorescent images were acquired on a Zeiss LSM900 confocal microscope from at least 10 randomly selected fields per condition.

### Flow cytometry analysis

Flow cytometry was performed using a Cell Cycle and Apoptosis Analysis Kit (Beyotime, Shanghai, China) as described by the manufacturer. Briefly, GBM cells (1 × 10^5^ cells per well) were seeded on 6-well plates and cultured at 37 °C and 5% CO_2_ for 24 h. Cells were treated with 0 or 100 nM CuB. After 24 h, cells were digested by 0.25% trypsin and diluted with 50 μL of PBS. And then, 500 μL of PI was added and protected from light at 37 °C for 30 min. The cell cycle was detected by flow cytometry (Beckman Coulter, United States).

### Bulk RNA-sequencing

Approximately 2 × 10^5^ cells were plated in a 6-well plate and cultured until reaching ∼80% confluence. Cells were then treated with either vehicle or CuB for 48 h. Total RNA was extracted using TRIzol Reagent following the manufacturer’s instructions. RNA quantity and purity were measured using a NanoDrop spectrophotometer, and RNA integrity was assessed using an Agilent 2,100 Bioanalyzer. Samples with RNA integrity number (RIN) ≥ 7.0 were used for library construction. RNA-seq libraries were prepared using a poly(A) mRNA sequencing (LC Sciences). Briefly, mRNA was purified from total RNA using oligo (dT) magnetic beads, fragmented, and reverse-transcribed to generate cDNA. After end repair, A-tailing, adaptor ligation, and PCR amplification, the final libraries were quantified, and fragment size distributions were examined using the Bioanalyzer. Libraries were pooled at equimolar concentrations and sequenced on an Illumina platform to generate paired-end 150-bp reads. Raw sequencing data were subjected to quality control using FastQC, and adapters and low-quality reads were removed using Trimmomatic. Clean reads were aligned to the human reference genome (GRCh38) using STAR. Gene-level read counts were quantified with feature Counts, and differential gene expression analysis was performed using DESeq2. Genes with adjusted p < 0.05 and |log_2_ fold change| ≥ 1 were considered significantly differentially expressed.

### Western blotting

Cells were collected and lysed in radioimmunoprecipitation (RIPA) buffer supplemented with protease and phosphatase inhibitors. Lysates were centrifuged at 4 °C for 15 min at 12,000 rpm, and the supernatant was collected. Protein concentrations were determined using the BCA Protein Assay Kit (Thermo Fisher Scientific). Equal amounts of protein (50 µg per sample) were separated on 10%–15% SDS-PAGE gels and transferred onto PVDF membranes (Millipore). Membranes were blocked with 5% skim milk in TBST for 2 h at room temperature, followed by incubation with primary antibodies overnight at 4 °C. The following primary antibodies were used: anti-GADD45α (1:1000), anti-PCNA (1:1000), anti-cleaved caspase-3 (1:1000), anti-CyclinB1 (1:1000), anti-p-CDK 1 (1:1000), anti-CDK 1 (1:1000), anti-p-Rb (1:1000), anti-E2F1 (1:1000) and anti-GAPDH (1:5000). After washing in TBST, membranes were incubated with HRP-conjugated secondary antibodies (1:5000) for 2 h at room temperature. The bands were visualized using an enhanced chemiluminescence (ECL) detection reagent (Yeasen, Shanghai, China) and imaged with a Tanon chemiluminescence imaging system (Tanon, Shanghai, China).

### Immunohistochemistry and histological staining

Mouse brain tumor tissues and kidneys tissues were fixed in 4% PFA, embedded in paraffin, and sectioned at a thickness of 4 μm. For Ki67 staining, sections were subjected to antigen retrieval in citrate buffer (pH 6.0), followed by quenching of endogenous peroxidase with 3% H_2_O_2_ for 10 min. Tissues were blocked with 5% goat serum and incubated overnight at 4 °C with specific primary antibodies. Detection was performed using an HRP-conjugated secondary antibody and DAB substrate, and nuclei were counterstained with hematoxylin.

For H&E staining, the paraffin-embedded tissue sections were deparaffinized in xylene and rehydrated through a descending graded alcohol series. The sections were then stained with hematoxylin solution for 5 min to visualize the nuclei, rinsed with tap water, and differentiated briefly. Subsequently, the sections were counterstained with eosin solution for 1–2 min to visualize the cytoplasm. Following dehydration through an ascending graded alcohol series and clearing in xylene, the slides were mounted with neutral balsam and examined under a light microscope to evaluate overall morphological structures and potential pathological lesions.

For TUNEL staining, apoptotic cells were detected using a commercial TUNEL detection kit according to the manufacturer’s instructions. After labeling, sections were counterstained with DAPI and mounted for imaging.

For immunostaining, slides were permeabilized and blocked for 2 h at room temperature in a buffer containing 10% goat serum and 0.3% Triton X-100 in PBS. Sections were then incubated overnight at 4 °C with primary antibodies against GADD45α or PCNA. After three PBS washes, tissues were incubated with appropriate fluorophore-conjugated secondary antibodies and DAPI for 30 min at room temperature. Fluorescent images were acquired using a Zeiss LSM900 confocal microscope (Carl Zeiss, USA).

### Quantitative RT-PCR

Total RNA from LN229 and U87 GBM cells were extracted using TRIzol reagent (Sigma Aldrich) following the standard protocol. RNA purity and concentration were determined by spectrophotometry, and 1 µg of total RNA was reverse-transcribed into cDNA using the PrimeScript™ RT Reagent Kit (Takara). Quantitative real-time PCR was performed on an Applied Biosystems StepOnePlus system using SYBR Green PCR Master Mix (Thermo Fisher Scientific). Gene expression levels were calculated using the 2^−ΔΔCT^ method, with GAPDH serving as the internal normalization control. Each reaction was performed in triplicate, and melting-curve analysis was conducted to verify amplicon specificity. Quantitative PCR (qPCR) primers used in this study were: human GADD45α Forward: 5′-AGAGCAGAAGACCGAAAGGA-3′and Reverse: 5′-CAG​GAT​CAG​GGT​GCT​CTT​TC-3′; human PCNA Forward: 5′-CGCTGCGACCGCAATTTTAG-3′and Reverse: 5′-AGG​AGT​GGA​GTG​GCT​TTG​GAA-3′; human GAPDH Forward: 5′-GAAGGTGAAGGTCGGAGT-3′and Reverse: 5′-GAA​GAT​GGT​GAT​GGG​ATT​TC-3′.

### Stereotaxic GBM cell transplantation

Stereotaxic implantation of GBM cells was performed using 5–6-week-old BALB/c nude mice. Mice were anesthetized with isoflurane and immobilized in a stereotaxic apparatus (RWD Life Science). Stable luciferase-expressing LN229 cells (LN229-luc cells, 5 × 10^5^ cells, 10 μL) were slowly injected into the striatum using the following coordinates relative to bregma: AP +1.0 mm, ML −2.0 mm, DV −3.0 mm. Cell suspension was delivered at a rate of 0.2 μL/min, and the injection needle was kept in place for an additional 5 min to prevent reflux before being withdrawn at 0.5 mm/min. Mice were monitored daily after implantation. Animals were euthanized upon development of neurological deficits, signs of distress, or moribund condition, in accordance with institutional animal care guidelines.

### *In vivo* bioluminescence analysis

Tumor progression *in vivo* was monitored using bioluminescence imaging (BLI). Luciferase-expressing GBM cells were implanted intracranially as previously described above (Gao et al., 2026; Zhong et al., 2024). For each imaging session, mice were anesthetized with isoflurane and administered d-luciferin (150 mg/kg, intraperitoneally) to serve as the luciferase substrate. After injection, mice were placed in a light-tight imaging chamber, and bioluminescent signals were acquired approximately 10–12 min later using an IVIS imaging system (PerkinElmer) equipped with Living Image software. Photon flux (photons/sec) from regions of interest (ROIs) was quantified to assess intracranial tumor burden. Baseline bioluminescence values were recorded prior to treatment randomization to ensure comparable initial tumor sizes across groups. During the observation period, luminescence measurements were normalized to baseline values to calculate relative tumor growth.

### *In vivo* toxicity assessment and serum biochemical analysis

To evaluate the *in vivo* safety and potential systemic toxicity of CuB, healthy mice were administered with CuB at the effective therapeutic dosage (0.5 mg/kg) or an equal volume of vehicle control following the same administration regimen as the orthotopic tumor model. Body weights of the mice were monitored and recorded regularly. At the end of the experimental period, blood samples were collected via eyeball extirpation into non-heparinized tubes. The blood samples were allowed to clot at room temperature for 30 min, followed by centrifugation at 3,000 × g for 15 min at 4 °C to separate the serum. To assess hepatic and renal functions, the serum levels of alanine aminotransferase (ALT), aspartate aminotransferase (AST), blood urea nitrogen (BUN), and creatinine (Cre) were quantitatively measured using commercially available diagnostic kits (Nanjing Jiancheng Bioengineering Institute, Nanjing, China) according to the manufacturer’s instructions.

### Statistical analysis

All statistical analyses were performed using GraphPad Prism 7 (GraphPad Software, San Diego, CA, USA). Survival data were analyzed using Kaplan–Meier survival curves and compared by the log-rank (Mantel-Cox) test. Comparisons among multiple groups were conducted using one-way or two-way analysis of variance (ANOVA), followed by Bonferroni’s *post hoc* test where appropriate. Data are presented as the mean ± SEM from at least three independent biological replicates. Differences were considered statistically significant at *P* < 0.05.

## Results

To evaluate the cytotoxic effects of CuB in human GBM, LN229 and U87 cells were exposed to various concentrations of CuB (0, 0.01, 0.1, 1, 10, and 100 μM) for 48 h. CCK-8 assays demonstrated a dose-dependent reduction in cell viability, with IC_50_ values of 1.323 μM for LN229 and 1.158 μM for U87 (Figure 1A). Consistently, colony formation assays showed that CuB treatment reduced clone area by ∼65% in LN229 and ∼70% in U87 compared with control group (Figures 1B,C). EdU incorporation assays further confirmed a significant inhibition of DNA synthesis, with EdU-positive cells decreasing from 78% ± 4.6% to 22% ± 2.8% in LN229 and from 54% ± 5.6% to 13% ± 3.1% in U87 (Figures 1D,E). Cell motility was also impaired by CuB. Wound healing assays demonstrated a 70% reduction in migration, and transwell invasion assays showed a 65% decrease in invading cells (Figures 1F–I). Furthermore, to address the clinical heterogeneity of GBM and evaluate the effects of CuB on therapeutically resistant subpopulations, we assessed patient-derived GSCs. CuB treatment markedly reduced the number and size of GSC neurospheres and significantly decreased stem-cell frequency in an extreme limiting dilution assay (ELDA) in a dose-dependent manner (Supplementary Figure S1). Collectively, these findings indicate that CuB robustly attenuates the malignant phenotype of GBM cells and stem-like populations.

**FIGURE 1:**
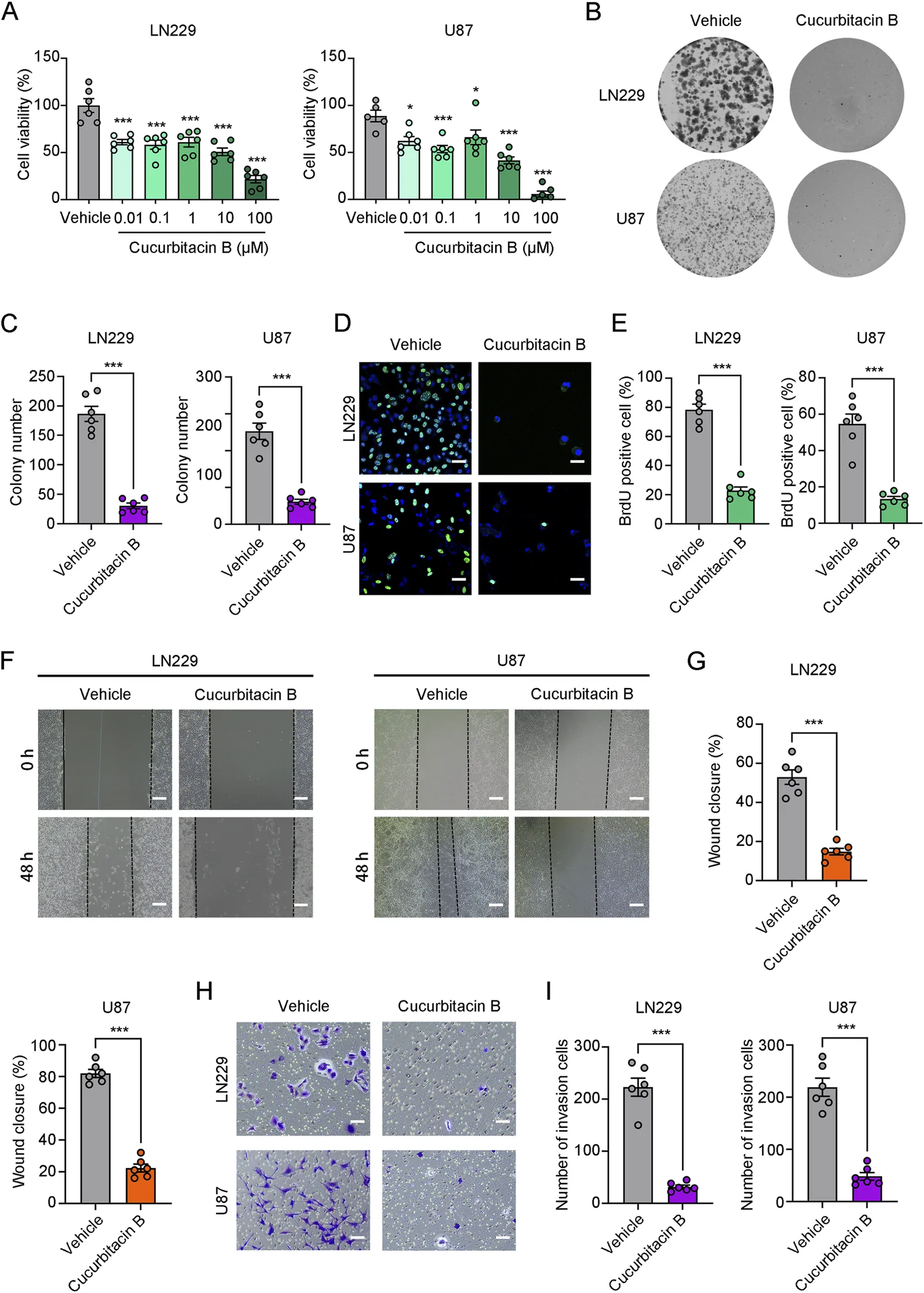
CuB inhibits the malignant progression of GBM cells *in vitro*. **(A)** Dose-dependent effects of CuB on LN229 and U87 cell viability assessed by CCK-8 assay after 48 h of treatment. IC50 values were 1.323 μM (LN229) and 1.158 μM (U87). **(B,C)** Representative images **(B)** and quantification **(C)** of colony formation in GBM cells treated with 100 nM CuB. **(D,E)** EdU incorporation assay showing reduced DNA synthesis following CuB exposure **(D)**; quantification of EdU-positive cells shown in **(E) (F,G)** Wound healing assays demonstrating CuB-induced inhibition of migration. **(H,I)** Transwell assay showing decreased invasion in CuB-treated GBM cells. Scale bar: 100 μm, or 200 μm. The data are represented as means ± SEM from at least three independent biological replicates. Statistical significance was determined one-way ANOVA with post-hoc testing, *P < 0.05, ***P < 0.001 vs. vehicle group.

To explore the molecular mechanisms underlying the inhibitory effects of CuB on GBM cells, LN229 and U87 cells were treated with 100 nM CuB for 24 h and subjected to bulk RNA sequencing. Differential gene expression analysis identified a total of 1,154 significantly altered genes following CuB treatment, including 515 upregulated genes and 639 downregulated genes (adjusted *p* < 0.05, |log_2_ fold change| ≥ 1) (Figures 2A,B).

**FIGURE 2:**
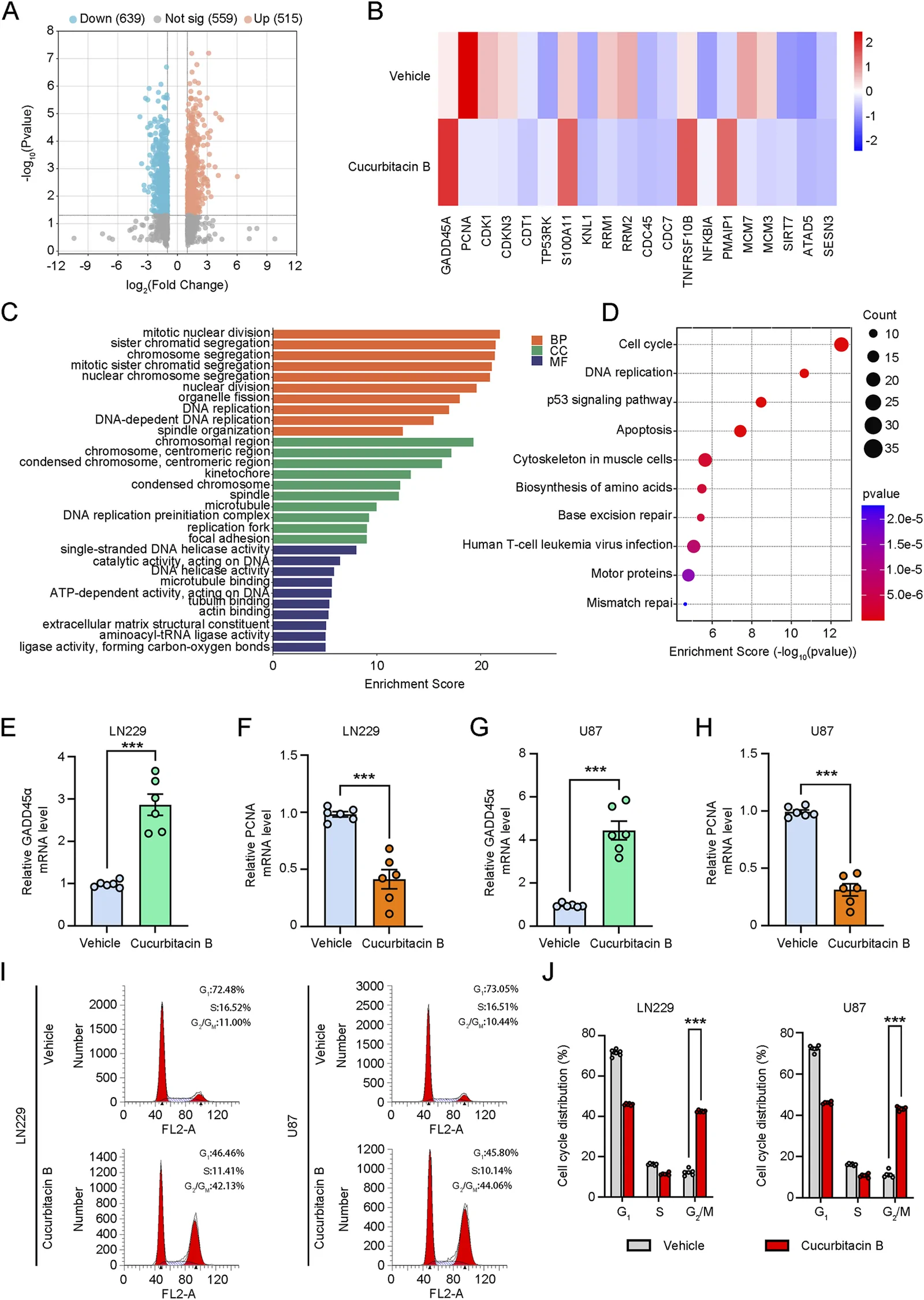
CuB induces GADD45α expression and G2/M cell-cycle arrest in GBM cells. **(A)** Volcano plot showing differentially expressed genes in GBM cells treated with 100 nM CuB for 24 h. **(B)** Heatmap shows gene-expression profiles after CuB treatment. **(C)** GO enrichment analysis of CuB-regulated genes highlighting DNA replication, cell-cycle, and mitotic pathways. **(D)** KEGG pathway analysis showing significant enrichment of cell-cycle and DNA replication pathways. **(E–H)** qPCR analysis of GADD45α and PCNA mRNA expression in CuB-treated LN229 **(E,F)** and U87 cells **(G,H) (I,J)** Flow cytometry showing G2/M-phase accumulation in LN229 **(I)** and U87 **(J)** cells following 24 h of CuB (100 nM) exposure. Data are presented as mean ± SEM from at least three independent biological replicates. Statistical significance was performed using one-way ANOVA with post-hoc testing, ***P < 0.001 vs. vehicle group.

To further investigate the biological processes affected by CuB, functional enrichment analyses were conducted. Gene Ontology (GO) analysis revealed that CuB-regulated genes were significantly enriched in biological processes associated with cell-cycle regulation, DNA replication, and mitotic progression (Figure 2C). Consistently, Kyoto Encyclopedia of Genes and Genomes (KEGG) pathway analysis showed that the cell cycle and DNA replication pathways were among the most significantly enriched pathways following CuB exposure (Figure 2D).

Among the differentially expressed genes, Growth Arrest and DNA Damage–Inducible Alpha (GADD45α) was markedly upregulated, whereas PCNA-a key component of the DNA replication machinery-was significantly downregulated. To validate these transcriptomic findings, quantitative PCR analysis was performed in LN229 and U87 cells. The results showed that GADD45α mRNA expression was significantly increased ∼3-fold after 12 h of CuB treatment, while PCNA mRNA decreased by ∼60% in both LN229 or U87 GBM cells, respectively (Figures 2E–H).

Given the critical roles of GADD45α in stress signaling and cell-cycle regulation, we next examined whether CuB treatment affects cell-cycle progression. Flow cytometry analysis demonstrated that CuB exposure resulted in a pronounced accumulation of GBM cells in the G2/M phase, accompanied by a corresponding reduction in the G0/G1 population, indicating a CuB-induced G2/M cell-cycle arrest (Figures 2I,J).

Collectively, these findings suggest that CuB disrupts GBM cell-cell-cycle progression, with GADD45α upregulation mediating PCNA suppression and replication inhibition, providing a mechanistic link between CuB exposure and the observed anti-proliferative effects.

To determine whether the anti-tumor effect of CuB is mediated through GADD45α, LN229 and U87 cells with stable GADD45α knockdown were generated using lentiviral shRNA (LV-GADD45α shRNA). Knockdown efficiency was approximately 80% reduction as confirmed by Western blot or RT-qPCR (Figures 3A,B; Supplementary Figure S2A). Functional assays demonstrated that GADD45α knockdown partially rescued CuB-induced inhibition of migration and invasion. Specifically, migration recovery increased from ∼30% to ∼85%, and invasion from ∼35% to ∼80% relative to vehicle-treated controls (Figures 3C,D). Similarly, colony formation and EdU incorporation assays further demonstrated that GADD45α knockdown restored proliferative capacity in CuB-treated cells (Figures 3E,F).

**FIGURE 3:**
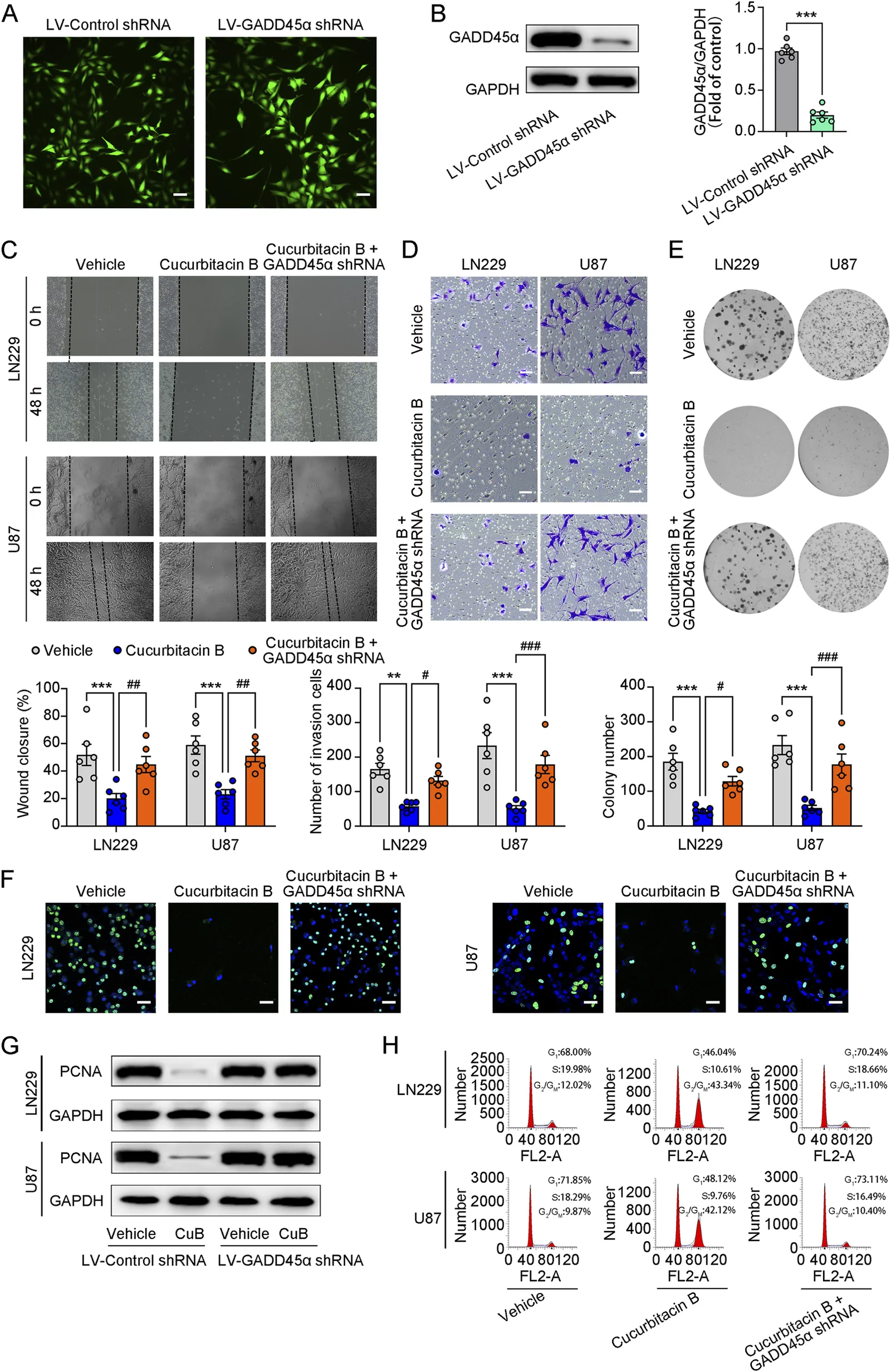
GADD45α knockdown reverses the antitumor effects of CuB in GBM cells. **(A,B)** Representative confocal images **(A)** and western blot bands **(B)** confirming efficient GADD45α knockdown in LN229 and U87 cells transduced with LV-GADD45α shRNA or LV-Control shRNA virus (titer, 1.00E + 10 PFU/mL, MOI = 20). **(C,D)** wound healing **(C)** and transwell invasion **(D)** assays showing partial rescue of CuB-induced inhibition of migration and invasion following GADD45α knockdown. **(E,F)** Colony formation **(E)** and EdU incorporation assays **(F)** demonstrating restoration of proliferative capacity in CuB-treated cells after GADD45α knockdown. **(G)** Representative western blot bands showing reversal of CuB-induced PCNA suppression in GADD45α knockdown GBM cells. **(H)** Flow cytometry of cell-cycle distribution. Scale bar: 100 or 200 μm. Data are presented as mean ± SEM from at least three independent biological replicates. Statistical significance was determined one-way ANOVA with post-hoc testing, **P < 0.01, ***P < 0.001 vs. vehicle group or LV-Control shRNA group; ^#^ *P* < 0.05, ^##^ *P* < 0.01, ^###^ *P* < 0.001 vs. Cucurbitacin B group.

At the molecular level, CuB-induced downregulation of PCNA protein expression was substantially reversed in GADD45α-deficient cells (Figure 3G). Flow cytometry further showed that CuB-induced G2/M arrest was alleviated by GADD45α knockdown, decreasing from 43% ± 2.2% to 11% ± 1.1% in LN229 cells, and from 42% ± 1.5% to 10% ± 0.8% in U87 (Figure 3H). Moreover, we also evaluated the key G2/M transition checkpoint proteins at the molecular level. CuB treatment markedly decreased Cyclin B1 expression and increased the inhibitory phosphorylation of CDK1; importantly, both alterations were fully reversed upon GADD45α knockdown (Supplementary Figures S3A-D). Furthermore, to address whether GADD45α is functionally required for CuB-induced apoptosis, we evaluated apoptotic markers. As expected, GADD45α silencing profoundly attenuated the CuB-induced accumulation of Cleaved Caspase-3 (Supplementary Figures S4A,B).

Collectively, these findings indicate that CuB restrains GBM progression in a GADD45α-dependent manner, predominantly through disruption of PCNA-regulated DNA replication and cell-cycle progression.

To investigate whether the anti-tumor effects of CuB are mediated through the GADD45α-PCNA axis, LN229 and U87 cells overexpressing GADD45α were generated using AAV vectors and verified by Western blotting or RT-qPCR (Figure 4A; Supplementary Figure S2B). Functionally, GADD45α overexpression markedly inhibited GBM cell proliferation (∼65% reduction in EdU-positive cells), migration (∼60% wound closure inhibition), and invasion (∼55% reduction in transwell invasion), phenocopying CuB treatment (Figures 4B–D).

**FIGURE 4:**
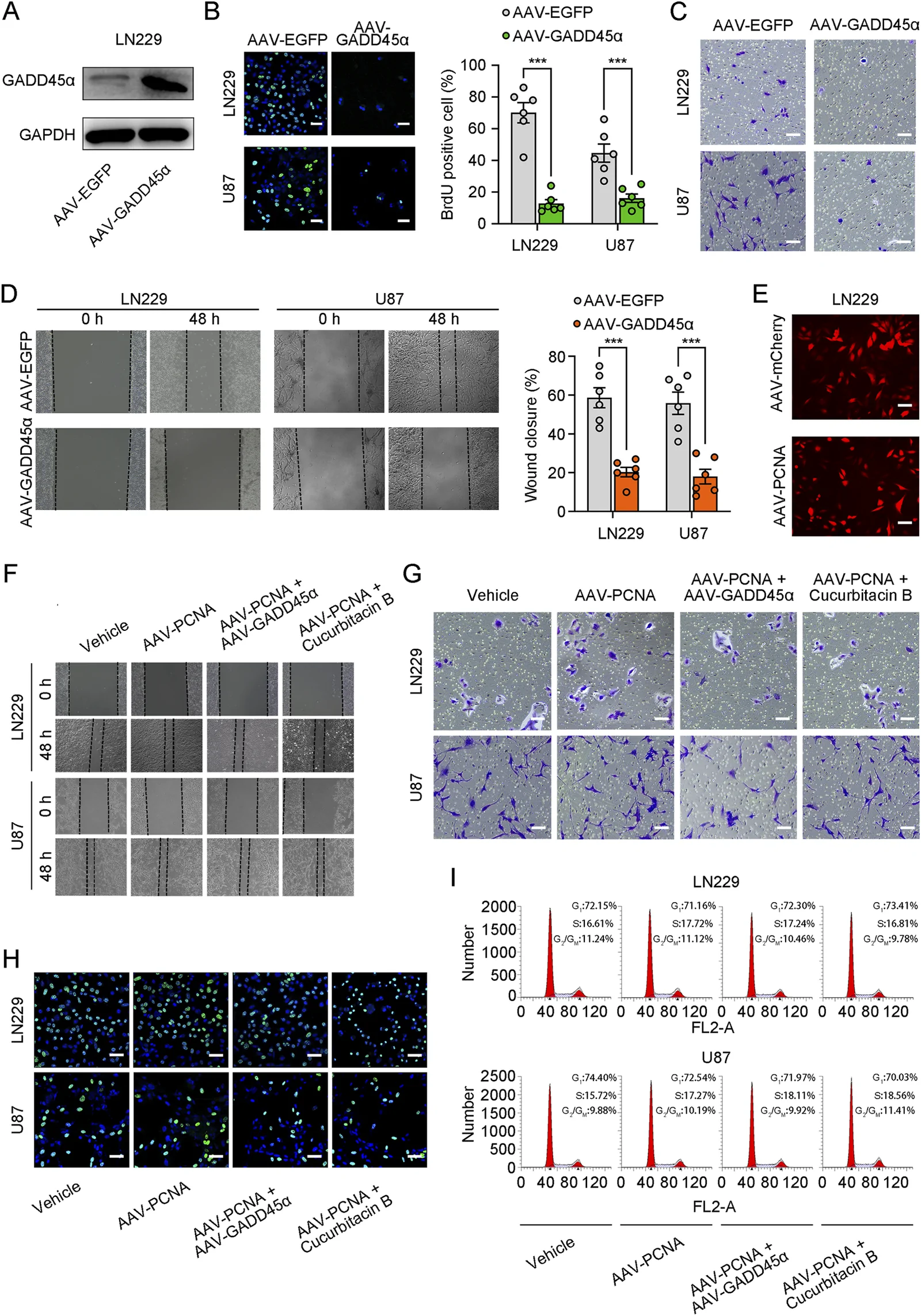
CuB inhibits GBM progression via the GADD45α-Rb/E2F1-PCNA signaling axis. **(A)** Representative western blot images confirming GADD45α overexpression in LN229 cells transduced with AAV-GADD45α or AAV-EGFP (titer, 1.00E + 13 PFU/mL, MOI = 10) for 72 h. **(B)** EdU assay showing inhibition of proliferation upon GADD45α overexpression. **(C)** Transwell assay showing decreased invasion in GADD45α-overexpressing cells. **(D)** Scratch assay assessing migration inhibition after GADD45α overexpression. **(E)** Representative confocal images confirming PCNA overexpression in GBM cells transduced with AAV-PCNA or AAV-mCherry (titer, 1.00E + 12 PFU/mL, MOI = 10) for 72 h **(F–H)** Functional assays demonstrating that PCNA overexpression rescues CuB- or GADD45α-induced suppression of migration **(F)**, invasion **(G)**, and proliferation **(H)**. **(I)** Flow cytometry showing alleviation of G2/M arrest by PCNA overexpression in LN229 and U87 cells. Scale bar: 100 or 200 μm. Data are presented as mean ± SEM from at least three independent biological replicates. Statistical significance was determined one-way ANOVA with post-hoc testing, ***P < 0.001 vs. AAV-EGFP group.

Given that PCNA was downregulated at the transcriptional level following CuB treatment (Figures 2G,H), we sought to elucidate the exact molecular mechanism connecting GADD45α induction to PCNA suppression. PCNA transcription is primarily driven by the E2F1 transcription factor, whose activity is directly restricted by the hypophosphorylated (active) Retinoblastoma (Rb) protein. As shown in Supplementary Figure S5, Western blot analysis revealed that either CuB treatment or exogenous GADD45α overexpression significantly decreased the phosphorylation of Rb (p-Rb Ser807) and simultaneously downregulated E2F1 protein levels. Importantly, the CuB-induced reductions of p-Rb and E2F1 were markedly reversed upon GADD45α knockdown. These molecular findings provide a crucial mechanistic link, demonstrating that GADD45α-induced cell-cycle arrest maintains Rb in a hypophosphorylated state, which in turn sequesters E2F1 and silences the transcription of PCNA.

To functionally validate whether this GADD45α-mediated suppression of PCNA is ultimately responsible for the observed anti-tumor effects, we performed rescue experiments. Immunofluorescence staining and molecular analyses confirmed efficient PCNA overexpression following AAV-PCNA transduction for 72 h (Figure 4E; Supplementary Figure S2C-G). Functionally, restoring PCNA expression significantly counteracted the suppressive effects of either GADD45α overexpression or CuB treatment on GBM cell migration, invasion, and proliferation (Figures 4F–H). Furthermore, flow cytometry analysis confirmed that PCNA overexpression successfully alleviated the G2/M cell-cycle arrest induced by GADD45α or CuB treatment (Figure 4I).

Collectively, these data establish a comprehensive mechanistic cascade, demonstrating that CuB attenuates GBM progression by activating GADD45α, which sequentially modulates the Rb/E2F1 axis to suppress PCNA-driven DNA replication.

To determine whether the anti-tumor effects of CuB are mediated via GADD45α *in vivo*, LN229-luc cells were stereotactically implanted into the striatum of immunocompromised mice. Daily intraperitoneal (*i.p.*) administration of CuB (0.5 mg/kg) markedly suppressed intracranial tumor growth, as assessed by bioluminescence imaging (Figures 5A,B). Importantly, the tumor-suppressive effect of CuB was largely abolished in mice implanted with GADD45α-knockdown GBM cells, indicating that GADD45α is essential for CuB-mediated tumor inhibition *in vivo* (Figures 5A,B). Kaplan-Meier survival analysis further demonstrated that CuB significantly prolonged the lifespan of tumor-bearing mice, whereas GADD45α knockdown markedly shortened survival and eliminated the survival benefit conferred by CuB (Figure 5C).

**FIGURE 5:**
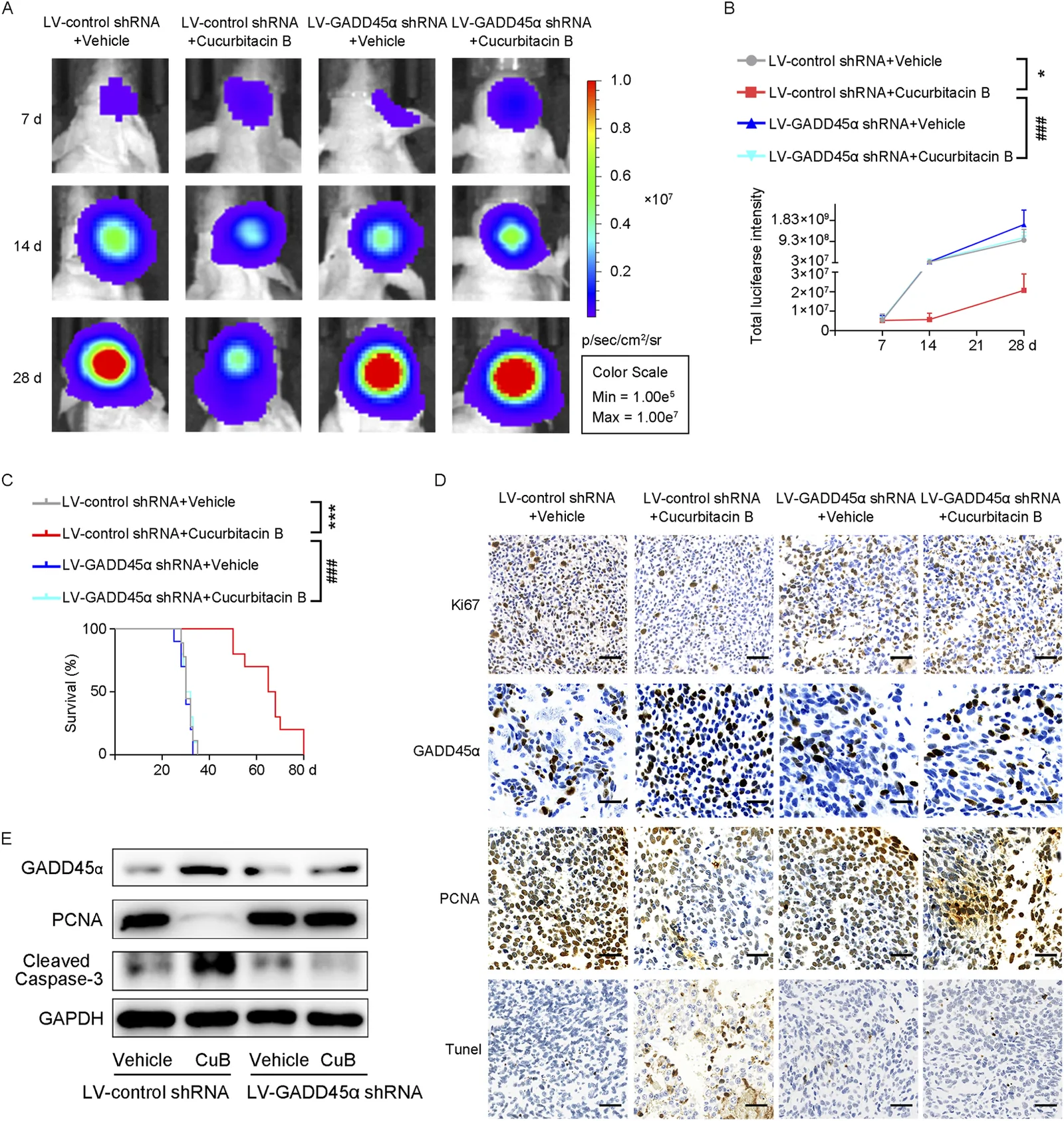
CuB suppresses tumor growth in an orthotopic GBM mouse model in a GADD45α-dependent manner. **(A,B)** Representative *in vivo* bioluminescent images **(A)** and quantitative luciferase signal intensities **(B)** in immunodeficient mice orthotopically implanted with 1 × 10^6^ LN229-luc cells transduced with LV-GADD45α shRNA or LV-control shRNA, and treated with CuB or vehicle (n = 10 per group). **(C)** Kaplan-Meier survival curves of mice bearing orthotopic GBM xenografts (n = 10 per group). **(D)** Representative immunohistochemical staining of Ki67, GADD45α, PCNA and TUNEL in tumor sections (n = 6 per group). **(E)** Representative western blot analysis of GADD45α, PCNA and cleaved caspase-3 expression in xenograft tissues (n = 4 per group). Scale bar: 100 μm. Data are presented as mean ± SEM from at least three independent biological replicates. Statistical significance was determined by one- or two-way ANOVA with post-hoc testing, *P < 0.05, ***P < 0.001 vs. LV-control shRNA + vehicle group; ^#^ *P* < 0.05, ^##^ *P* < 0.01, ^###^ *P* < 0.001 vs. LV-control shRNA + CuB group.

Immunohistochemical analyses revealed that CuB treatment prominently decreased the expression of Ki67 and PCNA while increasing GADD45α levels in tumor tissues; however, these effects were completely reversed by GADD45α knockdown (Figure 5D). Western blot analysis further confirmed the CuB-induced increase in GADD45α and reduction in PCNA expression. Moreover, cleaved caspase-3 levels were markedly elevated in CuB-treated xenografts, consistent with enhanced apoptosis (Figure 5E). TUNEL staining also revealed increased apoptotic cell death in CuB-treated tumors, whereas these effects were reversed by GADD45α knockdown *in vivo* (Figures 5D,E). Finally, to evaluate the translational potential and safety profile of CuB, we assessed *in vivo* toxicity in healthy mice. CuB administration at the therapeutic dosage did not induce significant body weight loss, impairment of hepatic or renal functions (ALT, AST, BUN, creatinine), or observable histological abnormalities in major organs (Supplementary Figure S6), suggesting a favorable safety profile.

Collectively, these findings demonstrate that CuB suppresses GBM growth and prolongs survival *in vivo* via a GADD45α-dependent mechanism, involving repression of PCNA-driven DNA replication and induction of apoptosis.

## Discussion

Cucurbitacin B, a highly bioactive tetracyclic triterpenoid derived from Cucurbitaceae plants, has attracted increasing attention for its potent anticancer activity across multiple tumor types. However, the mechanistic basis underlying its anti-GBM activity remained incompletely elucidated. In the present study, we demonstrate that CuB exerts strong anti-tumor activity in GBM by inducing the stress-response protein GADD45α, which subsequently suppresses PCNA-dependent DNA replication via the Rb/E2F1 transcriptional axis. Activation of this signaling cascade leads to profound G2/M phase cell-cycle arrest, inhibition of malignant progression, and induction of apoptosis both *in vitro* and *in vivo*. Importantly, these findings not only expand the current understanding of cucurbitacin pharmacology but also uncover a previously underexplored replication-stress-centered mechanism, highlighting the therapeutic potential of targeting the GADD45α-PCNA axis in GBM. underlying the anti-tumor activity of CuB.

Previous studies have demonstrated that cucurbitacins exert broad anti-tumor activity through multiple cellular mechanisms, including disruption of cytoskeletal architecture, activation of cellular stress responses, and modulation of oncogenic signaling pathway (Yin et al., 2025; Li et al., 2023). In GBM cells, CuB has been reported to rapidly induce morphological changes associated with actin cytoskeleton disruption, leading to impaired cell motility and reduced invasive capacity (Yin et al., 2008). Mechanistically, CuB promotes actin aggregation and formation of cofilin-actin rods through hyperactivation of cofilin mediated by the phosphatase slingshot homolog 1 (Zhang et al., 2014; Zhang et al., 2013). In addition, activation of the Gα13/RhoA/PKA signaling pathway has been shown to stimulate VASP phosphorylation and further enhance actin remodeling following CuB exposure (Zhang et al., 2013). Other studies have reported that cucurbitacins can also interfere with microtubule polymerization and cytoskeletal organization, thereby disrupting cell division and intracellular transport processes in tumor cells (Qin et al., 2018; Li et al., 2024; Touihri-Barakati et al., 2017; Lv et al., 2025; Xu et al., 2025). Collectively, these observations indicate that cytoskeletal perturbation represents an important cellular response to CuB treatment and contributes to its anti-tumor effects.

Cytoskeletal dynamics play a central role in the malignant behavior of glioblastoma. Actin-dependent structures such as lamellipodia and filopodia are critical for tumor cell migration and infiltration into surrounding brain tissue, which underlies the highly invasive nature of GBM (Giese et al., 2003; Sahai and Marshall, 2003). Recent studies further highlight that dysregulated actin remodeling contributes not only to tumor invasion but also to therapeutic resistance and adaptive stress responses in glioma cells (Yan et al., 2025). Alterations in cytoskeletal organization can modulate intracellular signaling, mechanotransduction (DuFort et al., 2011; Li and Wang, 2020), and cell-cycle progression (Hall, 2009), thereby influencing tumor sensitivity to anticancer agents (Masoumi et al., 2016; Lim et al., 2026). Although cytoskeletal perturbation is a well-established pharmacological hallmark of cucurbitacins, our transcriptomic and functional findings suggest that intrinsic stress-response programs—particularly DNA replication stress and cell-cycle checkpoint activation—crucially cooperate with, and perhaps drive, the terminal anti-tumor phenotype observed following CuB treatment.

Among the stress-responsive molecules induced by CuB, the growth arrest and DNA damage-inducible protein GADD45α emerged as a critical mediator of its anti-GBM activity. Members of the GADD45 family are key regulators of genomic stability, participating in DNA repair, replication fork control, chromatin remodeling, and activation of cell-cycle checkpoints (Palomer et al., 2024; Liebermann and Hoffman, 2008; Salvador et al., 2013; Tamura et al., 2012). Increasing evidence supports the tumor-suppressive role of GADD45 proteins in glioma biology. For example, GADD45 family members have been shown to modulate cellular responses to DNA damage and enhance the sensitivity of GBM cells to radiotherapy and chemotherapy (Hu et al., 2024; Wang H. H. et al., 2017). And GADD45G is identified as a favorable prognostic biomarker for glioma (Shen et al., 2025). Consistent with this paradigm, our results demonstrate that CuB strongly induces GADD45α expression in GBM cells. Intriguingly, our molecular evaluation of the G2/M checkpoint revealed that this GADD45α induction is accompanied by a profound downregulation of Cyclin B1 and an increase in the inhibitory phosphorylation of CDK1. Genetic depletion of GADD45α largely restored these cell-cycle regulators and abolished the CuB-induced suppression of GBM proliferation and invasion. These results firmly establish GADD45α as the central molecular link connecting CuB-induced cellular stress to robust cell-cycle arrest.

One major downstream effector within this replication-stress network is PCNA, a key sliding clamp that ensures DNA polymerase processivity and coordinates multiple protein complexes involved in DNA replication and repair (Strzalka and Ziemienowicz, 2011; Moldovan et al., 2007). Elevated PCNA expression is frequently observed in high-grade gliomas and is associated with increased proliferative capacity and poor clinical outcome (Korkolopoulou et al., 1994; Slade, 2018). While previous studies have reported that GADD45α can directly interact with PCNA to sterically hinder DNA synthesis (Chen et al., 1995; Zhan, 2005), our study uncovers a novel transcriptional regulatory mechanism mediated by CuB. We found that PCNA was heavily downregulated at the mRNA level following CuB treatment. Further investigation identified the classical Rb/E2F1 signaling cascade as the critical intermediate bridge. CuB-induced, GADD45α-mediated cell-cycle arrest maintains the Retinoblastoma (Rb) protein in a hypophosphorylated, active state. This active Rb tightly sequesters the E2F1 transcription factor, thereby shutting down the transcription of downstream E2F1-target genes, prominently including PCNA. Restoration of PCNA expression effectively rescued the inhibitory effects of CuB, validating this GADD45α-Rb/E2F1-PCNA transcriptional axis as a hallmark mechanism of CuB-induced replication stress.

Beyond replication blockade, our *in vivo* and *in vitro* findings further demonstrate that the prolonged replication stress initiated by the CuB/GADD45α axis ultimately primes tumor cells for apoptotic elimination. Increased TUNEL-positive cells and elevated cleaved caspase-3 levels were observed in CuB-treated xenografts, whereas GADD45α knockdown markedly attenuated these apoptotic responses. The dual impact of robust replication inhibition and the subsequent induction of apoptosis provide a comprehensive explanation for the substantial suppression of tumor growth and the prolonged survival observed in CuB-treated mice.

From a clinical and translational perspective, glioblastoma therapy is severely hindered by pronounced intratumoral heterogeneity and the existence of highly resistant GSCs. To ensure the broad therapeutic relevance of our findings, we confirmed that CuB robustly impaired the self-renewal and clonogenic capacities of patient-derived GSCs. This suggests that the GADD45α-mediated replication blockade is a conserved vulnerability across both bulk tumor cells and stem-like compartments. Furthermore, evaluating systemic safety and optimizing the therapeutic window is paramount for the clinical advancement of natural products. Our preliminary toxicological assessments revealed that CuB administration at the effective anti-tumor dosage was well tolerated in healthy mice, without causing hepatorenal dysfunction or noticeable histological lesions in major organs. While these initial safety profiles are highly encouraging, extensive pharmacokinetic studies and dosage optimization in larger pre-clinical models will be necessary to fully establish the therapeutic window of CuB.

## In conclusion

In conclusion, our study identifies a previously unrecognized mechanism by which cucurbitacin B suppresses glioblastoma progression. By inducing the stress-response protein GADD45α, CuB triggers cell-cycle arrest and modulates the Rb/E2F1 signaling pathway to transcriptionally downregulate PCNA-dependent DNA replication. This molecular cascade leads to replication arrest, inhibition of tumor proliferation and invasion, and the induction of apoptosis across diverse GBM cellular models. These findings not only expand the mechanistic understanding of cucurbitacin pharmacology and confirm its acceptable *in vivo* safety profile but also highlight the GADD45α–Rb/E2F1–PCNA axis as a highly promising therapeutic target for malignant gliomas.

## Data Availability

The original contributions presented in the study are publicly available. This data can be found here: GEO repository, accession number GSE347373.
