# Supplementary material for: Cucurbitacin B suppresses GBM progression by inducing GADD45α-Dependent PCNA downregulation and DNA replication arrest
Source: Front Pharmacol. 2026 Oct 14;17:1804502. doi: 10.3389/fphar.2026.1804502 (PMC13713883; doi:10.3389/fphar.2026.1804502)
Supplement: Supplementary file 1 [file DataSheet1.docx]

**Figure s1 CuB suppresses glioma stem cell self-renewal and clonogenicity *in vitro*.**

(A) Representative images of patient-derived glioma stem cell (GSC) neurospheres following treatment with increasing concentrations of CuB for the indicated duration. (B) Quantification of neurosphere number after CuB treatment. (C) Quantification of neurosphere diameter demonstrating impaired GSC proliferative capacity following CuB exposure. (D) Extreme limiting dilution assay (ELDA) analysis showing reduced stem-cell frequency in CuB-treated GSC cultures. Scale bar: 100 μm. Data are presented as mean ± SEM from three independent biological replicates. Statistical significance was determined using one-way ANOVA with post-hoc testing. ^**^*P* < 0.01, ^***^*P* < 0.001 vs. vehicle group.

**Figure S2. Validation of GADD45α and PCNA genetic manipulation efficiency in GBM cells.**

(A) RT-qPCR analysis confirming efficient knockdown of GADD45α mRNA expression in LN229 cells transduced with GADD45α shRNA compared with control shRNA cells. (B) RT-qPCR analysis validating GADD45α overexpression in LN229 cells following ectopic expression. (C–D) RT-qPCR analysis showing increased PCNA mRNA expression in LN229 and U87 cells following PCNA overexpression. (E–G) Representative Western blot images and quantitative densitometric analysis confirming elevated PCNA protein expression in LN229 and U87 cells after PCNA overexpression. Data are presented as mean ± SEM from three independent biological replicates. Statistical significance was determined using unpaired Student’s t-test, ^*^*P* < 0.05, ^**^*P* < 0.01 vs. LV-control shRNA group, or AAV-EGFP group, or AAV-mCherry group.

**Figure S3. CuB induces G2/M checkpoint activation through a GADD45α-dependent mechanism.**

(A) Representative Western blot images showing the expression of Cyclin B1, phosphorylated CDK1 (p-CDK1 Tyr15), total CDK1, and GAPDH in LN229 cells treated with vehicle, CuB, or CuB combined with GADD45α shRNA. (B-D) Quantitative densitometric analyses of Cyclin B1 (B), p-CDK1 Tyr15 (C), and total CDK1 (D) normalized to GAPDH. Data are presented as mean ± SEM from three independent biological replicates. Statistical significance was determined using one- or two-way way ANOVA followed by post-hoc testing. ^**^*P* < 0.01, ^***^*P* < 0.001 vs. vehicle group; ^##^*P* < 0.01, ^###^*P* < 0.001 vs. CuB-treated group; ns, not significant.

**Figure S4. GADD45α knockdown attenuates CuB-induced apoptotic signaling in GBM cells.**

(A) Representative Western blot images showing Cleaved Caspase-3 expression in LN229 and U87 cells treated with vehicle, CuB, vehicle + GADD45α shRNA or CuB + GADD45α shRNA. (B) Quantitative densitometric analysis of Cleaved Caspase-3 normalized to GAPDH. Data are presented as mean ± SEM from three independent biological replicates. Statistical significance was determined one-way ANOVA with post-hoc testing, ***P* < 0.01 vs. LV-control shRNA + vehicle group; ^##^*P* < 0.01 vs. LV-GADD45α shRNA + Cucurbitacin B group; ns, not significant.

**Figure S5. GADD45α mediates PCNA downregulation via the Rb/E2F1 signaling pathway.**

(A-D) Representative western blot analysis and quantitative densitometric analysis phosphorylated Rb (p-Rb Ser807), E2F1, PCNA and GAPDH in LN229 cells treated with vehicle, CuB, GADD45α OE, or CuB + GADD45α shRNA. Data are presented as mean ± SEM from three independent biological replicates. Statistical significance was determined one-or two-way ANOVA with post-hoc testing, ^*^*P* < 0.05, ^**^*P* < 0.01 vs. Vehicle group; ^##^*P* < 0.01, ^###^*P* < 0.001 vs. Cucurbitacin B group; ns, not significant.

**Figure S6. Preliminary *in vivo* safety evaluation of CuB in healthy mice.**

(A) Body weight changes in vehicle- and CuB-treated mice during the treatment period. (B) Representative H&E staining images of kidney tissues collected from vehicle- and CuB-treated mice. No obvious pathological abnormalities were observed following CuB administration. (C-F) Serum biochemical analyses of alanine aminotransferase (ALT) (C), aspartate aminotransferase (AST) (D), blood urea nitrogen (BUN) (E), and creatinine (Cre) (F) in vehicle- and CuB-treated mice. Scale bar: 100 μm. Data are presented as mean ± SEM from three independent biological replicates. Statistical significance was determined using unpaired Student’s t-test. ns, not significant.
